# Needle in a Radiologic Haystack: A Report of Duodenal Toothpick Perforation With Negative X-ray and CT Imaging

**DOI:** 10.7759/cureus.105006

**Published:** 2026-03-10

**Authors:** Jonathan Warren, Niresh Perera, Flor Lema, William Shyy

**Affiliations:** 1 Emergency Medicine, University of California Los Angeles David Geffen School of Medicine, Los Angeles, USA; 2 Emergency Medicine, Harbor University of California Los Angeles Medical Center, Torrance, USA; 3 Emergency Medicine, Veterans Affairs Greater Los Angeles Healthcare System, Los Angeles, USA

**Keywords:** emergency medical service, pocus (point of care ultrasound, point-of-care-ultrasound, prehospital, ultrasound

## Abstract

Foreign body ingestions are a common presentation in the emergency department. Many of these involve sharp or pointed objects, which may be challenging to detect when made of radiolucent material such as wood. While computed tomography (CT) increases sensitivity, perforation and mucosal injury are often diagnosed late because of false-negative scans. We report the rare case of duodenal toothpick perforation with negative radiographic imaging.

A 25-year-old male presented to urgent care after suspected accidental ingestion of a toothpick. The patient had no symptoms and no recollection of toothpick ingestion. After a normal X-ray, he was referred to the emergency department, where CT imaging of the neck, chest, and abdomen did not reveal any foreign body or secondary inflammatory changes. Gastroenterology was consulted and performed an endoscopy, identifying a 4-5 cm wooden toothpick embedded in and perforating the second portion of the duodenum. The foreign body was removed without complications.

This case highlights the importance of high clinical suspicion when encountering asymptomatic patients with potential foreign body ingestion of sharp radiolucent objects, even in the absence of radiographic or laboratory evidence of perforation. Endoscopic evaluation in these patients may be warranted to prevent delayed presentation and complications.

## Introduction

Foreign body ingestions are a common presenting complaint in emergency departments, occurring in over 100,000 cases per year [1]. Sharp, pointed objects are frequently accidentally or intentionally ingested and account for about 38% of all items [2]. About 44% of these pass without complication or intervention; however, 35% may be at risk for perforation [3]. Whenever ingestion is suspected, it is important to maintain a high clinical suspicion even with normal radiography, as the false-negative rate may approach 85% with wood and other organic ingestions [1]. With radiopaque materials, this false-negative rate is moderately improved and approaches 47%. The World Society of Emergency Surgery (WSES) recommends a secondary workup of possible foreign body ingestion with blood tests, including inflammatory markers, and computed tomography (CT), which has a sensitivity approaching 90%-100% [1]. In this case, we present a case of a male patient who accidentally ingested a toothpick, resulting in duodenal perforation despite a negative imaging workup with CT and X-ray.

## Case presentation

A 25-year-old male with a past medical history of remote diaphragmatic hernia repair and Nissen fundoplication presented to the emergency department for evaluation of possible foreign body ingestion. The patient reported that he was drinking water the previous day with a toothpick in his mouth. Although he experienced no specific abnormal sensation, he noted that the toothpick was missing after he finished drinking from his cup. He specifically denied any abdominal pain, foreign body sensation, nausea, vomiting, or chest pain.

The patient had previously received X-rays of his neck, chest, and abdomen at an outside urgent care facility, which did not identify any foreign body. Upon arrival at the emergency department, laboratory tests (complete blood count and basic metabolic panel) and CT scans of the neck, chest, and abdomen were performed to assess for a possible radiolucent foreign body. The CT scan was performed on a 64-slice scanner with 0.6-mm slices, without intravenous or oral contrast. The clinical indication was listed as "swallowed toothpick," and the emergency physician reviewed the results directly with the radiologist. Laboratory evaluation was within normal limits, with no evidence of leukocytosis or anemia. The CT scans demonstrated no evidence of a foreign body within the GI tract and no evidence of perforation.

**Figure 1:**
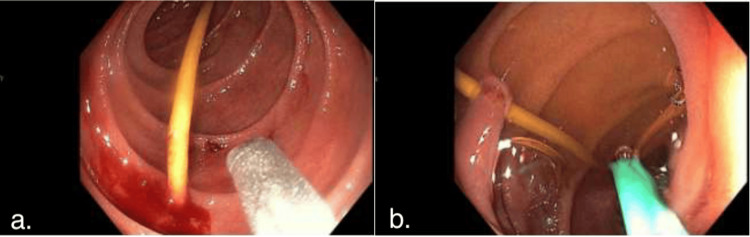
Endoscopic view of toothpick (a) Endoscopic visualization of an ingested toothpick through the second portion of the duodenum, resulting in perforations. (b) Endoscopic visualization of the ingested toothpick causing perforation through a fold of mucosa, resulting in two separate perforations.

The patient remained asymptomatic; however, based on the history, the case was reviewed with a gastroenterology specialist who recommended endoscopic evaluation. Endoscopy revealed a 4-5 cm long toothpick embedded within the proximal second portion of the duodenum (Figure 1).

**Figure 2:**
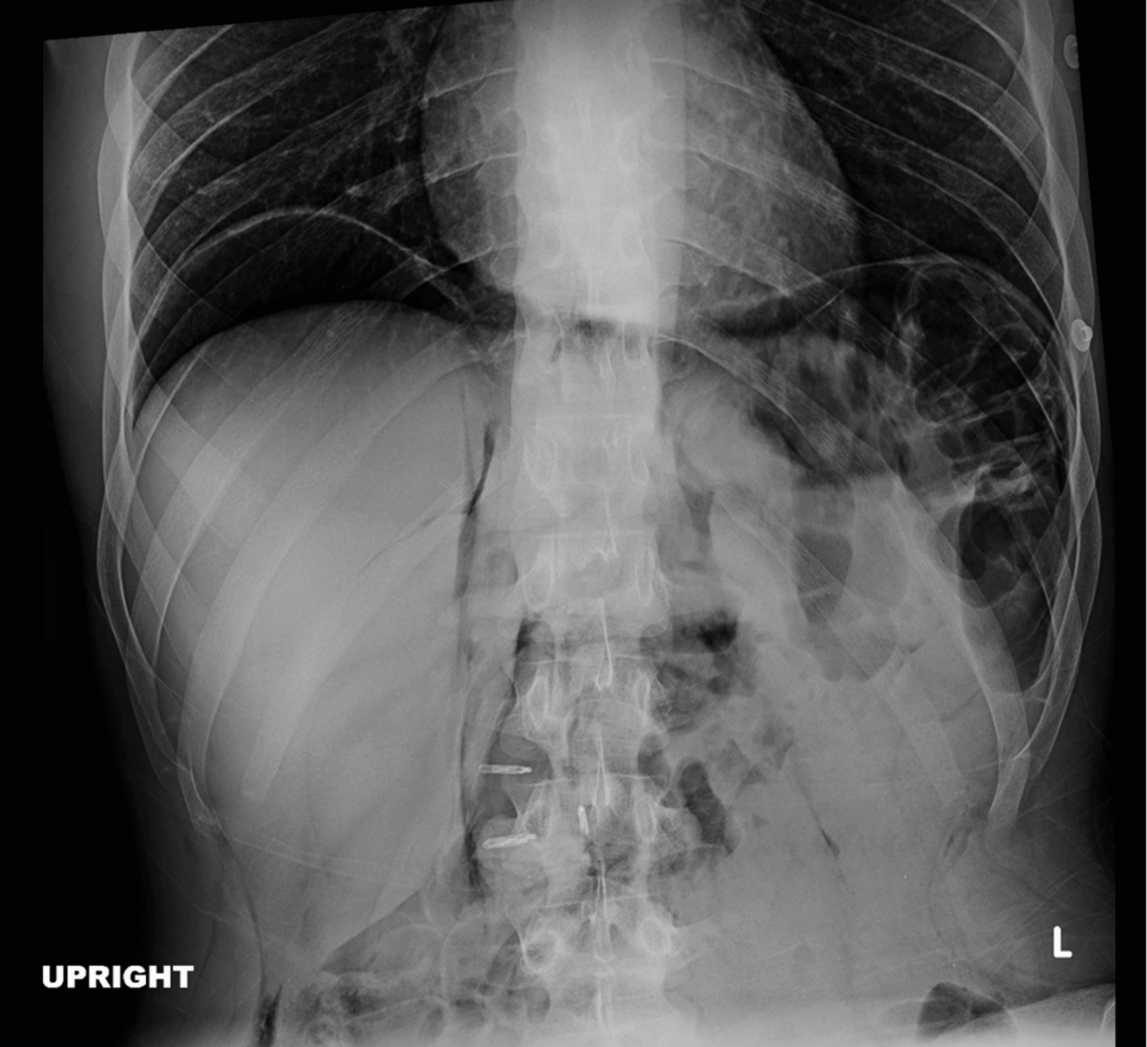
X-ray with diffuse pneumoperitoneum Upright abdominal X-ray obtained after esophageal and gastric insufflation during endoscopy showed diffuse pneumoperitoneum.

Upon removal of the toothpick, there were multiple small punctures closed by hemoclips. A post-procedural KUB X-ray demonstrated severe pneumoperitoneum concerning for perforation and insufflation during endoscopy (Figure 2).

Surgery was consulted for evaluation of pneumoperitoneum and agreed that it was likely a result of insufflation during endoscopy. They recommended bowel rest with serial abdominal examinations and KUBs for two days. Based on his reassuring examinations, no indication for urgent surgical intervention was identified. The patient was started on empiric antibiotics and a proton pump inhibitor to continue for a total of two weeks after discharge. The patient’s diet was slowly advanced, and he was ultimately discharged from the hospital without further complications.

## Discussion

Up to 43% of foreign body ingestions may be asymptomatic upon initial presentation [4]. In cases where ingestion of a foreign body is not confirmed, the diagnosis of a retained foreign body may be difficult to make, particularly due to the high false-negative rate of organic objects approaching 85% [1]. Given this, WSES Guidelines recommend further evaluation, specifically in equivocal cases with negative radiographs, with laboratory testing to include a complete blood count, C-reactive protein, blood gas, and lactate, as well as a CT scan. Abnormal findings on these exams may suggest underlying mucosal injury from a foreign body ingestion [5].

When foreign bodies are missed on the initial encounter, patients may not present until several days to weeks later, when perforation occurs and the patient develops abdominal pain [6]. Multiple case reports and series have demonstrated accidental ingestion of wooden toothpicks leading to perforation and presentations with abdominal pain, elevated inflammatory markers, and abnormal CT findings [6-8].

Our case differs from the existing literature in that our patient had a duodenal perforation but presented without abdominal pain, without elevated leukocytosis, and with a CT scan without secondary signs of perforation. Clinical suspicion for an ingested foreign body and subsequent complications must remain high. This case highlights the importance of guideline recommendations that endoscopy may be required in cases of a sharp ingested foreign body, even when not confirmed on definitive CT imaging [9].

Clinical pearls

Sharp radiolucent objects may be missed on CT scan. The absence of leukocytosis or other laboratory abnormalities does not exclude perforation. Early GI consultation and consideration of endoscopy are recommended even in asymptomatic patients with a concerning history.

## Conclusions

Recognition of risk and high clinical suspicion may help prevent delayed complications of perforation, such as abscesses or solid organ injury. Early consultation with a gastroenterology specialist is indicated for reports of sharp-object foreign body ingestions, even when patients are asymptomatic and have an otherwise normal evaluation.
